# T Cell Activation and Proinflammatory Cytokine Production in Clinically Cured Tuberculosis Are Time-Dependent and Accompanied by Upregulation of IL-10

**DOI:** 10.1371/journal.pone.0065492

**Published:** 2013-06-18

**Authors:** Marcos Vinícius da Silva, Amanda A. Figueiredo, Juliana R. Machado, Lúcio C. Castellano, Patricia B. D. Alexandre, Rafael F. Oliveira, Gladstone E. L. Faria, Sanívia A. L. Pereira, Denise B. R. Rodrigues, Virmondes Rodrigues

**Affiliations:** 1 Laboratory of Immunology, Department of Biological Sciences, Triângulo Mineiro Federal University, Uberaba, Minas Gerais, Brazil; 2 Department of Technical School of Health, Paraíba Federal University, João Pessoa, Paraíba, Brazil; 3 Laboratory of Biopathology and Molecular Biology, University of Uberaba, Uberaba, Minas Gerais, Brazil; 4 Uberaba Municipal Department of Health, Uberaba, Minas Gerais, Brazil; University of Cape Town, South Africa

## Abstract

**Background:**

Th1 cytokines are essential for the control of *M. tuberculosis* infection. The role of IL-10 in tuberculosis is controversial and there is an increasing body of evidence suggesting that the relationship between Th1 cytokines and IL-10 is not as antagonistic as it was first believed, and that these cytokines may complement each other in infectious diseases.

**Methods:**

The present study evaluated the activating capacity of CD4+ and CD8+ T cell repertoire in response to antigen stimulation through the expression of CD69 using Flow Cytometry, as well as the functionality of PBMCs by determining the cytokine profile in patients with active tuberculosis and in clinically cured patients after in vitro stimulation using ELISA. Treated patients were subdivided according to time after clinical cure (<12 months or >12 months post-treatment).

**Results:**

We observed that T cell activation was higher in TB-treated patients, especially CD8+ T cell activation in TB-Treated >1 year. Th1 cytokines were significantly higher in TB-Treated, and the levels of IFN-γ and TNF-α increased continuously after clinical cure. Moreover, IL-10 production was significantly higher in cured patients and it was also enhanced in cured patients over time after treatment. Th17, Th2 and Th22 cytokines showed no statistically significant differences between Healthy Donors, Active-TB and TB-Treated.

**Conclusions:**

This study describes a scenario in which potentiation of CD4+ and CD8+ T cell activation and increased Th1 cytokine production are associated with the clinical cure of tuberculosis in the absence of significant changes in Th2 cytokine production and is accompanied by increased production of IL-10. In contrast to other infections with intracellular microorganisms, this response occurs later after the end of treatment.

## Introduction

Tuberculosis continues to be a global public health problem with an enormous social impact. About 2 billion people are currently infected with *Mycobacterium tuberculosis* [1]. The protective immune response against *M. tuberculosis* is highly dependent on the interaction between T cells and macrophages, and disturbances in the mechanisms of cell-mediated immunity, particularly HIV infection and immunomodulatory therapy, are related to the reactivation of infection [2].

Th1 cytokines are essential for the control of *M. tuberculosis* infection. Together, IFN-γ and TNF-α activate macrophages, potentiating their microbicidal mechanisms [3], inducing mechanisms of autophagy involved in the clearance of *M. tuberculosis* [4], and maintaining the structural integrity of granulomas [5]. In the latter context, TNF-α blocking leads to loss of granuloma structure and to reactivation of the disease [6]. On the other hand, it is still difficult to determine whether strong Th2 or Treg responses are the cause or consequence of the development of tuberculosis in humans [7], [8]. The role of IL-10 in tuberculosis is also controversial. Production of IFN-γ is not increased in IL-10^−/−^ knockout mice infected with *M. tuberculosis* [9], although these animals have long-term uncontrolled inflammatory responses and progression of the disease [10]. There is an increasing body of evidence suggesting that the relationship between Th1 cytokines and IL-10 is not as antagonistic as it was first believed, and that these cytokines may complement each other in infectious diseases [11].

In human tuberculosis, the development of a functional T cell repertoire during active disease and after the establishment of clinical cure is still not fully defined. The aim of the present study was to evaluate T cell-activating capacity and cytokine production *in vitro* after stimulation with crude antigen extract of *M. bovis* during active human pulmonary tuberculosis and after treatment, as well as to correlate the findings with the time after clinical cure.

## Methods

### Patients

Peripheral blood samples were collected from 25 patients with active pulmonary tuberculosis, from 24 patients with clinically cured pulmonary tuberculosis (TB-Treated group) and from 20 healthy donors. Treated patients were subdivided according to time after clinical cure: <12 months (n = 11) and >12 months (n = 13) after the end of treatment. None of the TB-treated patients had any sign or symptom indicating that active disease developed after the end of the treatment. As control group, we selected healthy individuals without history of tuberculosis (pulmonary or extrapulmonary) but with a positive tuberculin test, located in the same microregion and which also comply with the exclusion criteria. All the participants in this study were recruited from the city of Uberaba, in the state of Minas Gerais, in the southeast region of Brazil. Brazil is one of the 22 high-burden countries that collectively account for about 80% of the TB cases in the world. In the state of Minas Gerais, an average 6,085 cases/year occurred in the last six years, with an incidence coefficient of 23 cases/100,000 inhabitants, the 4^th^ higher TB burden in the country. The city of Uberaba had 73 confirmed cases in 2012 [12], [13].

The diagnosis of tuberculosis was based on clinical, radiographic and laboratory findings and it was performed by the Municipal Department of Health team in Uberaba, Minas Gerais state, Brazil. Patients with a diagnosis of pulmonary tuberculosis were immediately referred for specific chemotherapy according to the regimen recommended by the Brazilian Ministry of Health. Blood was collected from patients with active disease within a maximum period of 21 days after the beginning of the treatment in order to reduce the impact of therapy on the parameters studied (median of 20 days; IQR 7). HIV-infected patients with or without clinical disease, transplant patients, patients using immunosuppressive drugs, patients with chronic alcoholism, malnourished patients, and patients with any known cause of immunosuppression were excluded from the study. All the participants recruited were previously vaccinated with BCG. The clinical characteristics of the patients are shown in Table 1.

**Table 1:** Clinical characteristics of the patients.

Group			Gender		Age (years)	Time after clinical cure (months)
	n	%	Male	Female	Mean (SE)	Mean (SE)
Healthy Donors	20	29.0%	8 (40%)	12 (60%)	38.65 (2.44)	-
Active tuberculosis	25	36.2%	11 (46.9%)	14 (53.1%)	42.58 (2.64)	-
TB-treated	24	34.8%	12 (50%)	12 (50%)	47.2 (3.14)	18.29 (3.72)
TB-treated<1 year	11	15.9%	5 (45.8%)	6 (54.6%)	48.54 (4.66)	6.26 (0.93)*
TB-treated>1 year	13	18.8%	7 (54.2%)	6 (46.2%)	45.8 (4.37)	29.22 (17.24)*

TB-treated: clinically cured tuberculosis patients; SE: standard error.

* p<0.05, unpaired t-test

### Ethics Statement

This study was approved by the Ethics Committee of Federal University of Triângulo Mineiro, Uberaba, Minas Gerais state, Brazil, and all participants signed the Free and Informed Consent Form.

### Isolation and culture of peripheral blood mononuclear cells

Peripheral blood mononuclear cells (PBMCs) were isolated by Ficoll-Hypaque density gradient centrifugation (GE Healthcare, Uppsala, Sweden) at 400× *g* at 21°C for 20 min. The cells were then resuspended in RPMI 1640 medium (GE Healthcare) containing 50 mM Hepes buffer (Gibco, Grand Island, NY, USA), 5% inactivated fetal bovine serum (Gibco), 2 mM L-glutamine (Gibco), and 40 µg/mL gentamicin (Neoquímica, Anápolis, GO, Brazil) to a final concentration of 2×10^6^ cells/mL. PBMCs were cultured in 24-well microplates (Falcon, San Jose, CA, USA) in the presence of 4 µg/mL *M. bovis* antigen, or maintained in culture medium at 37°C in a 5% CO_2_ atmosphere. Moreover, PBMCs from 5 Healthy Donors, 4 TB-treated patients and 4 Active-TB patients were cultured in the presence of 2 ug/mL phytohemagglutinin (PHA) for 48 hours. The cells were collected after 48 h for immunophenotyping, and the supernatants were collected after 48 and 120 h and stored at −70°C for the measurement of soluble mediators with ELISA. A plasma sample was also stored under the same conditions for later quantification of cytokines.

### Preparation of Mycobacterium bovis antigen

*Mycobacterium* antigens were extracted from *Mycobacterium bovis* (bacillus Calmette-Guérin, BCG), strain Moreau (Instituto Butantan, São Paulo, Brazil). The mycobacteria were first resuspended in 0.85 g NaCl at a concentration of 2×10^6^ bacteria/mL in accordance with the recommendations of Instituto Butantan. Next, the bacteria were incubated in a water bath at 90°C for 30 min and then autoclaved for 30 min. The cultures were centrifuged at 10,000× *g* at 4°C for 30 min and the supernatant (protein fraction) was collected and filtered through 0.22-µm Millipore filter (Molsheim, France). Protein concentration was quantified by the Bradford method (Pierce, Rockford, IL, USA) and the protein fraction was divided into aliquots and stored at −20°C.

### Analysis of T cell activation

For analysis of T cell activation, PBMCs cultured for 48 h were resuspended (5×10^5^ cells/mL) in Hank's medium (Sigma, St. Louis, MO, USA), washed three times (400× *g*, 4°C, 10 min), and incubated in Hank's medium supplemented with 10% inactivated human AB+ serum. After that, the samples were labeled with anti-CD4-PeCy5, anti-CD8-FITC and anti-CD69-PE antibodies or appropriate isotype controls (BD Pharmingen, San Diego, CA, USA). The cells were washed to remove excess antibodies, resuspended in 500 µL PBS containing 0.5% paraformaldehyde, and stored at 4°C in the dark until flow cytometry analysis. A FACSCalibur cytometer (Becton-Dickinson, Mountain View, CA, USA) was used for the acquisition of events (50,000 events/tube) and the data were analyzed using the Cell Quest program (Becton-Dickinson).

### Quantification of cytokines in plasma and cell culture supernatants

Concentrations of IL-4, IL-6, IL-10, IL-12, IL-13, IL-22, IFN-γ, TNF-α, LT-α, TGF-β (BD Pharmingen) and IL-17 (R&D Systems, Minneapolis, MN, USA) in culture supernatants were measured by ELISA using pairs of monoclonal antibodies, in accordance with the manufacturer's specifications. Briefly, high-affinity 96-well plates (Nunc, Roskilde, Denmark) were sensitized with cytokine-specific monoclonal antibodies, followed by blocking with PBS containing 2% BSA (Sigma). The supernatants and recombinant cytokines were then added, and the plates were incubated for 4 h at room temperature. The plates were washed and incubated with 1 µg/mL biotinylated anti-cytokine monoclonal antibody at 37°C for 2 h, followed by washing and incubation with alkaline phosphatase-conjugated streptavidin at 37°C for 2 h. The reaction was developed using disodium-p-nitrophenyl phosphate (Sigma) in diethanolamine buffer. Absorbance was read at 405 nm in a microplate reader (Bio-Rad, Hercules, CA, USA). Cytokine concentration was calculated using linear regression analysis of absorbance values obtained for the recombinant cytokines, and it was expressed as pg/mL. The sensitivity of the tests ranged from 2 to 20 pg/mL.

### Statistical analysis

Statistical analysis was performed using StatView software (version 4.57, Abacus Concept, Berkeley, CA, USA). Continuous variables with normal distribution are expressed as mean ± standard deviation and the variables without normal distribution are expressed as the median, with range and percentiles. For variables with normal distribution and homogeneity of variance, ANOVA test was used for comparison of three or more groups. Variables with no normal distribution or homogeneity of variance were analyzed using Kruskal-Wallis test to compare three or more groups. Post-hoc tests were conducted to evaluate pairwise differences between three or four groups, controlling for Type I error across tests by using the Bonferroni/Dunn test for multiple comparisons. Statistical differences between cytokine production in unstimulated and stimulated cultures were analyzed using Wilcoxon test. Differences were considered statistically significant when p<0.017 between 3 groups, and when p<0.008 between 4 groups, applying Bonferroni corrections for multiple comparisons. Correlation analyses were performed using the Spearman test, and were considered significant when p<0.05.

## Results

### Percentage of in vitro activated T cells after antigen stimulation

**Figure 1:**
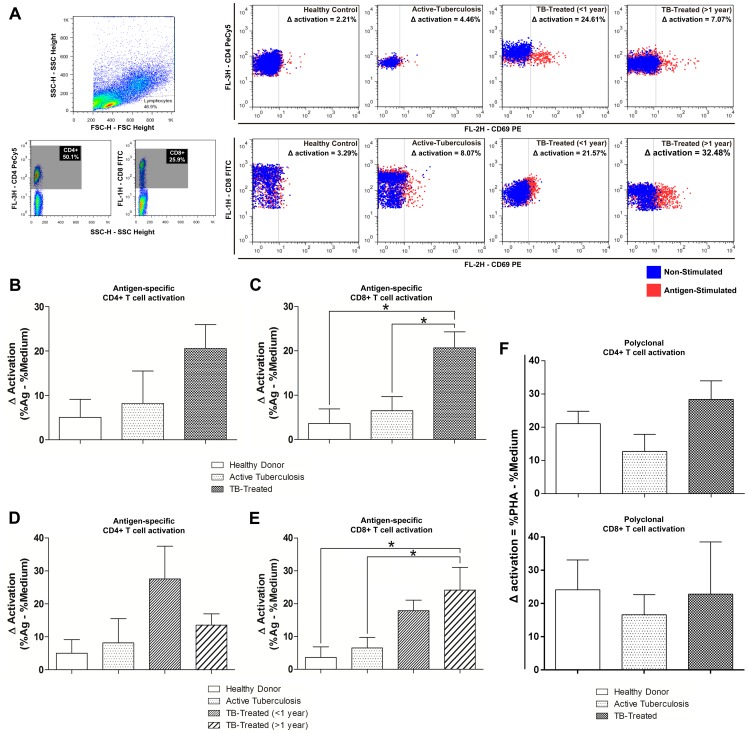
T cell activation in active and clinical cured tuberculosis. Variation in the activation of helper and cytolytic T cells in Healthy Donors, patients with active tuberculosis and clinically cured patients. **A**. Schematic representation of the gating strategy and determination of Δ activation of CD69+ cells in stimulated (4 µg/mL *M. bovis* antigen) and unstimulated (medium only) cultures. From left to right, T cells were separated based on FSC and SSC patterns. These cells were divided into CD4+ and CD8+ and the expression of CD69 was evaluated. Dot plots representative of each studied group are shown. Blue dots represent the staining for CD69 in unstimulated cultures, and red dots represent the stimulated cultures. The percentage of specific antigen activation was calculated by simple subtraction of the percentage of CD69+ cells in unstimulated cultures from the percentage observed in stimulated cultures and the Δ activation for each depicted dot plot is shown. The superior panel of dot plots represents the activation in CD4+, and the inferior panel represents the activation in CD8+. Dot plots representative of a single participant are shown. **B, C**. Comparison between Healthy Donors, Active-TB patients and TB-treated patients. **D, E**. Comparison between Healthy Donors, Active-TB and different times after clinical cure (TB-treated <1 year and TB-treated >1 year). **F**. Comparisons between Healthy Donors, Active-TB patients and TB-treated patients after culture in the presence of polyclonal stimulation– 2 ug/mL PHA (Polyclonal activation was calculated by simple subtraction of the percentage of CD69+ cells in unstimulated cultures from the percentage observed in PHA-stimulated cultures). Bars represent the mean and vertical lines the standard error. **B, C, F**. *p<0.0167: ANOVA test (followed by post-hoc Bonferroni test for multiple comparisons). **D, E**. *p<0.0083: ANOVA test (followed by post-hoc Bonferroni test for multiple comparisons).

The percentage of activated CD4+ and CD8+ T cells after specific stimulation was measured by the expression of CD69. CD69 is a molecule expressed in T cells after stimulation via TCR [14], [15], and, although it is an early activation marker which is expressed within minutes after cell activation, it is relatively stable, especially *in vitro* [16], [17]. Furthermore, the expression of CD69 in T cells after *in vitro* stimulation with mycobacterial antigens or antigens from the sites of infection indicates previous exposure to *M. tuberculosis* [18], [19]. Activated T cell phenotype was examined by the co-expression of CD4/CD69 (activated helper T cells) and CD8/CD69 (activated cytolytic T cells) markers. Cell activation is expressed as the change in the percentage of activated cells after antigen stimulation in comparison with the unstimulated culture (Δ activation), as illustrated in Figure 1A.

Both cell populations were activated after antigen stimulation, with higher Δ activation in TB-treated patients than in Active-TB patients or in Healthy Donors, and we observed a significant difference in CD8+ T cells between TB-Treated and Active-tuberculosis (p = 0.005) or Healthy Donors (p = 0.004) (Fig. 1B and 1C). Analysis of TB-treated patients according to time after clinical cure showed higher activation of CD4+ T cells in patients who had been treated more recently. CD4+ T cells returned to levels which were similar to those observed in patients with active disease over time after the end of treatment, but the result was not statistically significant (Fig. 1D). Activation of cytolytic T cells was increased in TB-treated patients <1 year, but the result was not statistically significant, and this increase was even higher in patients who had been cured more than 1 year ago in comparison with Active-TB patients or Healthy Donors (p = 0.004 and p = 0.003, respectively) (Fig. 1E). Moreover, we compared the percentage of CD4+ and CD8+ T cells activation after polyclonal stimulation using Phytohemagglutinin (PHA - 4 µg/ml). Phytohemagglutinin is a lectin that activates T cells by indirectly cross-linking the TCR because TCR-negative cells will not respond to these agents [20]. We did not observe any statistically significant differences between the groups (Fig. 1F).

### Production of Th1 cytokines in active tuberculosis and after treatment

**Figure 2:**
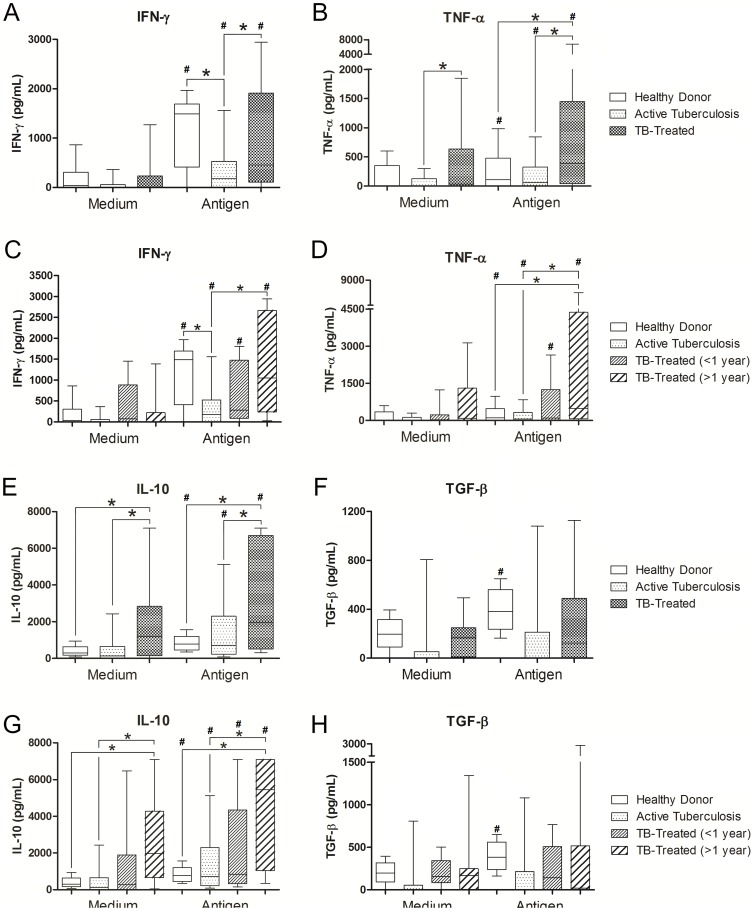
Th1 and T regulatory cytokines in active and clinical cured tuberculosis. Production of IFN-γ, TNF-α, IL-10 and TGF-β by PBMCs from Healthy Donors, patients with active tuberculosis and clinically cured patients in unstimulated (medium only) and stimulated (4 µg/mL *M. bovis* antigen) cultures. **A, B, E, F**. Comparison between healthy donors, patients with active tuberculosis and clinically cured patients (TB-treated). *p<0.0167: Kruskal-Wallis test (followed by post-hoc Bonferroni/Dunn test for multiple comparisons). **C, D, G, H**. Comparison between Healthy Donors, patients with active tuberculosis and different times after clinical cure (TB-treated<1 year and TB-treated>1 year). *p<0.0083: Kruskal-Wallis test (followed by post-hoc Bonferroni/Dunn test for multiple comparisons). Horizontal lines represent the median, bars represent 25–75 percentiles, and vertical lines represent 10–90 percentiles. #p<0.05 in unstimulated versus stimulated cultures (4 µg/mL *M. bovis* antigen), Wilcoxon test.

We evaluated cytokine production by PBMCs in culture supernatants in the absence or presence of antigen stimulation and cytokine concentrations were expressed as pg/mL. The levels of Th1 cytokines (IFN-γ and TNF-α) were significantly higher in the TB-Treated group than in the Active-TB group when antigen-stimulated culture supernatants were compared (p = 0.007 and p = 0.004, respectively). In addition, the production of IFN-γ was higher in Healthy Donors than in Active-TB patients (p = 0.006) (Fig. 2A and 2B). We did not observe statistically significant differences in the levels of IFN-γ and TNF-α in non stimulated cultures. When the treated group was analyzed according to time after clinical cure, IFN-γ and TNF-α levels were found to continuously increase after clinical cure. However, the difference was significant only in patients >1 year after the end of treatment who had significantly higher IFN-γ and TNF-α levels in antigen-stimulated culture supernatants than patients with active disease (p = 0.001 and p = 0.0002, respectively) (Fig. 2C and 2D). During the period after clinical cure, the group treated >1 year had significantly higher TNF-α levels than Healthy Donors (p = 0.0003), and compatible IFN-γ levels between these groups were observed. All the groups analyzed had a higher production of IFN-γ and TNF-α after antigenic stimulation in comparison with unstimulated cultures. Our results indicate that clinical cure of pulmonary tuberculosis is characterized by recovery in IFN-γ production capacity and by enhancement of TNF-α production. This increase in the production of IFN-γ and TNF-α was not accompanied by change in the production of other Th1-related cytokines such as IL-12 and LT-α (data not shown). The results suggest that the development of a Th1 profile in human tuberculosis occurs late after clinical cure.

### Production of IL-10 and TGF-β in active tuberculosis and after treatment

In addition to the increased production of Th1 cytokines (IFN-γ and TNF-α), IL-10 production was significantly higher in cured patients than Active-TB and Healthy Donors in unstimulated cultures (p = 0.002 and p = 0.001, respectively), and in antigen-stimulated cultures (p = 0.009 and p = 0.003, respectively) (Fig. 2E). Analysis of the TB-Treated group according to time after clinical cure showed that this increase was only significant in patients treated more than 1 year ago, and we observed significantly higher IL-10 levels in unstimulated and stimulted culture supernatants in comparison with Active-TB patients (unstimulated: p = 0.0006, stimulated: p = 0.003) or Healthy Donors (unstimulated: p = 0.0003, stimulated: p = 0.0001). All the groups had a significantly higher production of IL-10 in stimulated cultures in comparison with basal production (p<0.05 for all comparisons) (Fig. 2G). We did not observe statistically significant differences in the production of TGF-β, both basal and after stimulation (Fig. 2F and 2H). Only the healthy control group had a statistically significant difference between basal production and production after stimulation (p = 0.011) (Fig. 2H).

### Correlation of cytokine production in active tuberculosis and after treatment

**Figure 3:**
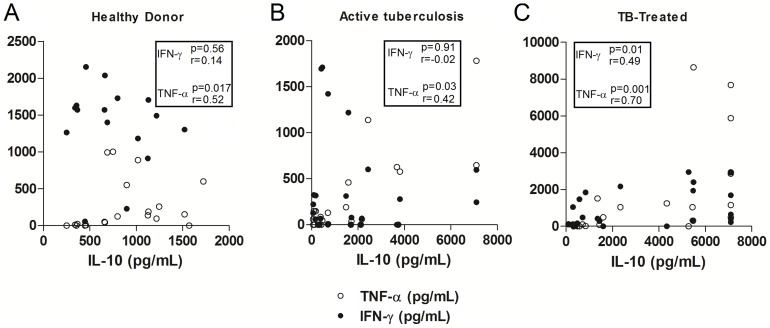
Correlation between IL-10 and Th1 cytokines in active and clinical cured tuberculosis. Correlation between IL-10 and IFN-γ or TNF-α levels produced by PBMCs from Healthy Donors (**A**), patients with active tuberculosis (**B**) and clinically cured patients (**C**) in stimulated cultures (4 µg/mL *M. bovis* antigen). Values of *p* and *r* determined using Spearman's correlation test.

In order to determine whether the higher expression of Th1 cytokines (IFN-γ and TNF-α) observed in the establishment of clinical cure was accompanied by an increased expression of regulatory cytokines, particularly IL-10, we tested the correlation between the production of these cytokines after antigen stimulation in patients with active disease and in clinically cured patients. No correlation between the production of IFN-γ and IL-10 was observed in Healthy Donors; however, there was a positive and significant correlation between TNF-α and IL-10 (p = 0.017, r = 0.52) (Fig. 3A). No correlation between the production of IL-10 and IFN-γ was observed in patients with active tuberculosis, although there was a significant positive correlation between IL-10 and TNF-α in stimulated cultures (p = 0.03, r = 0.42) (Fig. 3B). In contrast, a significant positive correlation between IL-10 and TNF-α (p<0.001, r = 0.70) and between IL-10 and IFN-γ (p = 0.01, r = 0.49) was observed in clinically cured patients (Fig. 3C).

### Th2, Th17 and Th22 axis in active and clinically cured tuberculosis

**Figure 4:**
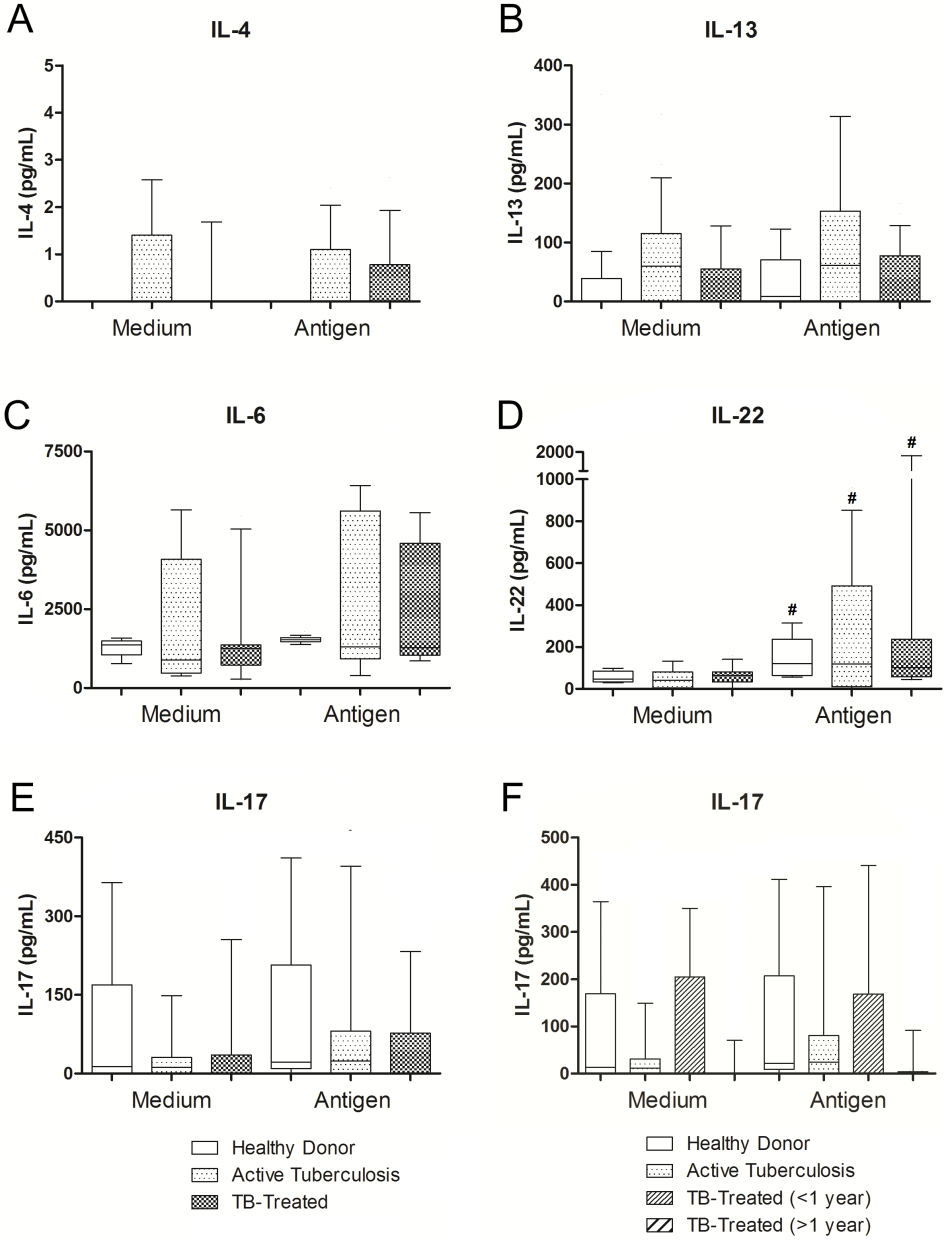
Th2, Th17 and Th22 cytokines in active and clinical cured tuberculosis. Production of IL-4, IL-13, IL-17, IL-6 and IL-22 by PBMCs from Healthy Donors, patients with active tuberculosis and clinically cured patients in unstimulated (medium only) and stimulated (4 µg/mL *M. bovis* antigen) cultures. **A, B, C, E, F**. Comparison between patients with active tuberculosis and clinically cured patients (TB-treated). **D**. Comparison between active tuberculosis and different times after clinical cure (TB-treated<1 year and TB-treated>1 year). Horizontal lines represent the median, bars represent 25–75 percentiles, and vertical lines represent 10–90 percentiles. #p<0.05 in unstimulated versus stimulated cultures (4 µg/mL *M. bovis* antigen), Wilcoxon test.

No significant differences in the production of IL-4 or IL-13 were observed between the Active-TB group, the TB-Treated group and the Healthy Donors under any of the culture conditions studied (Fig. 4A and 4B). No significant difference was observed when the time after clinical cure was taken into account (data not shown). We did not observe any differences between basal production and production after stimulation.

The production of IL-6 or IL-22 did not differ significantly between Active-TB, TB-Treated and the Healthy Donors under any of the culture conditions (Fig. 4C and 4D). There was also no difference when the time after clinical cure was taken into account (data not shown). Even though we did not observe any differences in IL-6 production between stimulated and unstimulated cultures, all the groups had a significantly higher antigen-specific production of IL-22. No significant differences in the production of IL-17 were observed between Active-TB patients, TB-treated patients or Healthy Donors, even though the patients after clinical cure seemed to tend to have lower levels than the healthy control patients in both cultures (Fig. 4E and 4F).

### Cytokine plasma levels in active and clinically cured tuberculosis

**Figure 5:**
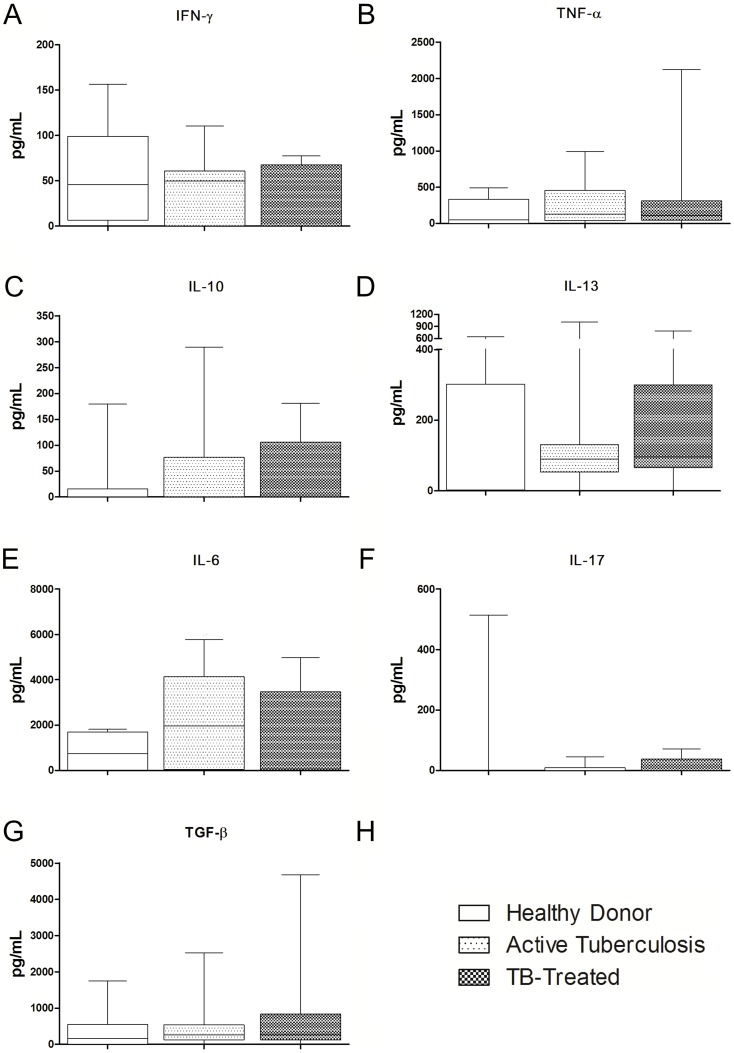
Cytokine plasma levels in active and clinically cured tuberculosis. Plasma levels of TNF-α, IFN-γ, IL-10, IL-4, IL-13, IL-6, IL-17, TGF-β and IL-22 in Healthy Donors, patients with active tuberculosis and clinically cured patients (**A–G**). Horizontal lines represent the median, bars represent 25–75 percentiles, and vertical lines represent 10–90 percentiles. *p<0.0167: Kruskal-Wallis test (followed by post-hoc Bonferroni/Dunn test for multiple comparisons).

In order to analyze whether the pattern of cytokine levels observed after PBMC culture was also observed regarding its systemic levels, we quantified plasma levels of TNF-α, IFN-γ, IL-10, IL-4, IL-13, IL-6, IL-17, TGF-β and IL-22 and compared Healthy Donors, Active-TB and TB-Treated. We did not observe any statistically significant difference in any of the cytokines analyzed (Fig. 5), whereby IL-4 and IL-22 did not have detectable levels.

## Discussion

The role of cell-mediated immunity in infection with *M. tuberculosis* has been reinforced over the last years either in experimental models or in studies of the human disease. The role of T cells and cytokines has been extensively investigated in the maintenance of latent infection, in its manifestation as active disease, or in resolution of infection and clinical cure (for a review, see Ref. 4). The present study analyzed the activating capacity of CD4+ and CD8+ T cell repertoire in response to antigen stimulation through CD69 expression, as well as the functionality of PBMCs by determining the cytokine profile produced after *in vitro* stimulation in patients with active tuberculosis, in clinically cured patients and in healthy donors living in the same region. The results showed that clinical cure of tuberculosis is characterized by a greater T-cell-activating capacity in response to antigen stimulation, a difference that is more pronounced in cytolytic T cells. This observation confirms previous reports suggesting deficient T cell activation in patients with active tuberculosis [21], [22]. However, it remains unclear whether this impairment is a direct consequence of the infection, or whether it is directly associated with latent period termination. Studies suggest that the induction of regulatory T cell subpopulations (CD4+/CD25+/Foxp3+) may impair activation of the specific T cell repertoire (both CD4+ and CD8+) in active tuberculosis [23]. A recent study using an experimental model supports the concept that the maintenance of latent infection depends on the activating capacity of anti-*M. tuberculosis* cells and disturbances in this process favor the growth of microorganism and potential reactivation of infection [24]. Furthermore, clinical cure of tuberculosis has been associated with an increment in the activation potential of CD4+ and CD8+ T cells, in which both subpopulations become important for the production of cytokines such as IFN-γ and cytokines, maintaining the integrity of granulomas. Although CD4+ T cells are activated early after the end of treatment, this *in vitro* antigen-specific activation decreases over time after clinical cure. In contrast, the activation of cytolytic T cells increases after clinical cure. In fact, in pulmonary tuberculosis the *in situ* production of IFN-γ associated with clinical cure seems to be related to the close collaboration between helper and cytolytic T cells [25].

The importance of IFN-γ in resistance to infection with *M. tuberculosis* has been extensively studied and demonstrated in experimental models and in human diseases. In this respect, mice have shown to be more susceptible to *M. tuberculosis* infection in situations in which IFN-γ production is compromised [2], [26]. Moreover, mutations in the IFN-γ gene and its receptors in humans are associated with severe disease [27], [28]. Studies indicate a decrease in the *in vitro* IFN-γ production by PBMCs in active disease [29], and animal models have provided evidence that reduced IFN-γ production results in a massive inflow of neutrophils into the lung and in the subsequent tissue damage seen in tuberculosis [30]. On the other hand, an increase in the production of IFN-γ after specific therapy has been reported, although the levels were lower than those observed in patients with latent tuberculosis [22], [29]. Similar to the increase in IFN-γ, we demonstrated an increase in the production of TNF-α over time after the clinical cure of tuberculosis, but in TB-treated patients the increase was significantly higher than in the healthy control group. In fact, previous studies have shown a decreased production of TNF-α in patients with active disease or with multidrug-resistant tuberculosis [31], [32]. In contrast, Sahiratmadja et al. [29] observed a decrease in the production of TNF-α during the course of anti-tuberculosis therapy, specifically at the end of treatment, but the authors did not evaluate post-therapy changes. This concomitant occurrence of enhanced production of cytokines classically related to cell-mediated immunity, to activation of macrophages and to maintenance of granuloma integrity seems to be extremely rational, especially when considering that non-sterilizing cure is frequent in tuberculosis.

The increased production of IFN-γ and TNF-α in clinically cured patients was not accompanied by increased production of IL-12. The results regarding IL-12 levels in active tuberculosis, especially after PMBCs culture, are controversial [33], [34]. However, this fact does not rule out a role of this cytokine in the modulation of IFN-γ levels, since local differences in the effects and production of IL-12 are observed in affected organs and lymph nodes [35] which cannot be detected directly in *in vitro* assays. Furthermore, the production of LT-α, a cytokine which is also related to the maintenance of granuloma integrity in different diseases [5], [36], did not differ between patients with active disease and clinically cured patients. In agreement with experimental studies, we suggest that in tuberculosis LT-α only acts as an auxiliary to TNF-α in granuloma maintenance, and deficiency of the latter cytokine has a greater impact on disease reactivation [37], [38]. Studies comparing the production of LT-α in patients with active tuberculosis and after treatment, or its variation over time after clinical cure, are scarce.

The present study showed that the development of a Th1 response, characterized by IFN-γ and TNF-α production, occurs late with the observation of this response in patients clinically cured for more than 12 months. Although studies evaluating the production of these cytokines over time after clinical cure are scarce, Zhang et al. [39] reported an increase in IFN-γ levels over time. Similarly, Hirsch et al. [40] observed that IFN-γ production was enhanced in cured patients only over time post-treatment. This slow development of a Th1 response in human tuberculosis differs from that seen in tegumentary leishmaniasis, another infection in which a Th1 response is associated with cure and is established immediately after treatment [41]. On the other hand, Caldas et al. [42] suggested that increased production of IFN-γ associated with clinical cure of visceral leishmaniasis occurs late and is likely to be related to systemic reduction of IL-10, but not to antigen-specific reduction. In our study we showed that changes in IFN-γ, TNF-α and IL-10 occur mainly in an antigen-specific way, without changes in plasma levels. In fact, previous studies showed that serum cytokines appear to only poorly reflect peripheral blood cellular cytokine production observed *in vitro* [43], [44]. Several factors may contribute to these differences, for instance: low plasma levels of most cytokines, differences in counter-regulatory mechanisms found *in vivo* and *in vitro*, influence of cell culture surface on the capability of activating patients' PBMCs, as well as the time of culture that favors higher levels *in vitro* than *ex vivo* [43], [44], [45].

In the present study, the process of clinical cure characterized by the potentiation of Th1 cytokine (IFN-γ and TNF-α) production was accompanied by higher levels of IL-10, a cytokine which is classically implied in the regulation of the production of these cytokines [46]. Our results point to an increase that is dependent on the immune system and that was triggered by the infection, since, similar to TNF-α, increased IL-10 after treatment has significantly higher levels than those observed in the healthy group. This was demonstrated by the significant positive correlation of IFN-γ and TNF-α levels with IL-10 in stimulated culture supernatants of clinically cured patients. Recent studies have shown an increase in IL-10 in some infectious diseases. This increased production is responsible for a reduction in the deleterious effects of the Th1 cytokine-mediated inflammatory reaction without impairing the clearance of infectious agents [11]. In infections in which the control of the infectious agent is mediated by a strong Th1 response, such as infection with *Listeria monocytogenes*, *Trypanosoma cruzi* and respiratory influenza virus, the concomitant presence of IL-10 may prevent the tissue damage associated with Th1 responses [47], [48], [49]. Similarly, in experimental models the protective response to infection with *Toxoplasma gondii* is associated with the production of IFN-γ; however, in the absence of IL-10, infected animals succumb earlier as a result of uncontrolled immunopathology, although parasite control seems to be intact [50], [51]. *In vitro* studies have shown that Th1 cells could be the source of this IL-10 in the control of infections with intracellular pathogens and these cells maintain their IFN-γ-producing function intact, and consequently activate macrophages or IL-10-producing Treg cells (CD4+CD25+) both in infection with *L. major* or with *M. tuberculosis* [23], [52], [53]. Furthermore, IL-10 may potentiate inflammatory mediators, especially in an environment rich in IFN-γ [54]. Within this scenario, the strong positive correlation of IFN-γ and TNF-α with IL-10 production during the establishment of clinical cure of pulmonary tuberculosis seems to contribute to the control of the microorganism without inducing exacerbated tissue damage. In contrast to previous views, the capacity to produce IL-10 has not only been demonstrated in Th2 or Treg cells [55], [56], but also in Th1, Th9, Th17 and CD8+ T cells [46], [57], [58], [59], [60]. The concomitant increase in Th1 cytokines and IL-10 may reflect the induction of distinct populations, which act together in the establishment of clinical cure of tuberculosis, or of IFN-γ/IL-10 double-producing populations. Furthermore, our results point to an increased basal production of IL-10 *in vitro* in TB-Treated >1 year, yet without differences in its plasma values. The basal production of IL-10 by PBMCs *in vitro* was previously reported under other conditions [44], [61], [62], [63], [64]. The increased basal production of cytokines *in vitro* in patients with pulmonary tuberculosis was previously reported for IFN-γ, with lower values after antigenic stimulation [65]. We particularly believe that the increased production of IL-10 after clinical cure of pulmonary tuberculosis occurs due to memory T cells [66], [67]. The presence of these cells might explain the differences in healthy controls or active-TB patients. Actually, a previous study shows an increase in effector memory T cells during treatment for tuberculosis [66]. As previously discussed, the conditions of the cultures themselves may act as a stimulus for the basal production of cytokines, by different cell populations, or even APCs carrying antigens in the bloodstream [45]. Furthermore, we cannot rule out the induction of other related mechanisms, such as the ability of macrophages to secrete cytokines *in vitro*, which may undergo changes during clinical cure. Further studies investigating these hypotheses, probably in a single cell level, would be extremely important to clarify both the findings on basal production and antigen-specific production observed in our study.

Analysis of Th2 cytokines revealed no significant differences between the groups of patients studied. An association between increased IL-4 production and progression of tuberculosis has been demonstrated in experimental models [7], although the absence of this cytokine does not influence the susceptibility to disease [68]. Recently, the participation of Th2 cytokines in the susceptibility to tuberculosis has been associated with the inhibition of autophagy, compromising antigen presentation, clonal expansion of T cells, and granuloma organization [69], [70]. On the basis of previous evidence and of the present findings, we may hypothesize that the influence of Th2 cytokines is more impressive in patients with active tuberculosis since they present a reduced capacity to produce Th1 cytokines. On the other hand, in the establishment of clinical cure, the increase in IFN-γ and TNF-α reduces the impact of Th2 cytokines on the anti-*M. tuberculosis* response.

The levels of cytokines related to the induction and/or maintenance of the Th17 axis did not differ between patients with active and cured tuberculosis, although both groups produced less IL-17 than healthy donors and post-treatment time seems to be related to the virtual disappearance of IL-17. This finding is in consonance with recent studies associating IL-17 production and the induction of neutrophil accumulation in the lung with the aggravation of damage induced by *M. tuberculosis* infection [71], with high IFN-γ production reducing this migration and associated lesions [30]. On the other hand, Jurado et al. [72] found an association between an increase in the number of IFN-γ+IL17+ double-producing T cells and poor prognosis in active tuberculosis. Further studies using *Mycobacterium*-specific T cell lines can help clarify the presence and true role of T cells producing IL-17 alone or in combination with IFN-γ in the susceptibility to disease aggravation or establishment of clinical cure.

This study describes a scenario in which the potentiation of CD4+ and CD8+ T cell activation and increased production of Th1 cytokines are associated with the clinical cure of tuberculosis in the absence of significant changes in the production of Th2 cytokines. Furthermore, the development of this Th1 response is accompanied by an increase in IL-10 production. In contrast to other infections with intracellular microorganisms, this response occurs later after the end of treatment. The results suggest that therapeutic intervention in tuberculosis is the initial trigger for recovery of the protective immune response in infected patients, thus reducing bacillary load and potentiating Th1 immune response. However, recovery of the capacity to respond to myobacterial antigens, including cell activation and the production of cytokines that mediate resistance to infection, is slow and is clearly established only after one year of specific treatment.
